# Eucalypts face increasing climate stress

**DOI:** 10.1002/ece3.873

**Published:** 2013-11-12

**Authors:** Nathalie Butt, Laura J Pollock, Clive A McAlpine

**Affiliations:** 1 ARC Centre of Excellence for Environmental Decisions and School of Biological Sciences, The University of QueenslandSt. Lucia, Queensland, 4072, Australia; 2 National Environmental Research Program, School of Botany, The University of MelbourneMelbourne, Victoria, 3010, Australia; 3 National Environmental Research Program and School of Geography, Planning and Environmental Management, The University of QueenslandSt. Lucia, Queensland, 4072, Australia

**Keywords:** Climatic stress, eucalypts, forest ecosystems, rainfall seasonality, range shifts.

## Abstract

Global climate change is already impacting species and ecosystems across the planet. Trees, although long-lived, are sensitive to changes in climate, including climate extremes. Shifts in tree species' distributions will influence biodiversity and ecosystem function at scales ranging from local to landscape; dry and hot regions will be especially vulnerable. The Australian continent has been especially susceptible to climate change with extreme heat waves, droughts, and flooding in recent years, and this climate trajectory is expected to continue. We sought to understand how climate change may impact Australian ecosystems by modeling distributional changes in eucalypt species, which dominate or codominate most forested ecosystems across Australia. We modeled a representative sample of *Eucalyptus* and *Corymbia* species (*n* = 108, or 14% of all species) using newly available Representative Concentration Pathway (RCP) scenarios developed for the 5th Assessment Report of the IPCC, and bioclimatic and substrate predictor variables. We compared current, 2025, 2055, and 2085 distributions. Overall, *Eucalyptus* and *Corymbia* species in the central desert and open woodland regions will be the most affected, losing 20% of their climate space under the mid-range climate scenario and twice that under the extreme scenario. The least affected species, in eastern Australia, are likely to lose 10% of their climate space under the mid-range climate scenario and twice that under the extreme scenario. Range shifts will be lateral as well as polewards, and these east–west transitions will be more significant, reflecting the strong influence of precipitation rather than temperature changes in subtropical and midlatitudes. These net losses, and the direction of shifts and contractions in range, suggest that many species in the eastern and southern seaboards will be pushed toward the continental limit and that large tracts of currently treed landscapes, especially in the continental interior, will change dramatically in terms of species composition and ecosystem structure.

## Introduction

### Changing climates

As indicated by many northern hemisphere-based studies, changes in climate will have major consequences for the distribution and functioning of ecosystems, and their biota (Rosenzweig et al. 2008; Chen et al. 2011; Nogues-Bravo and Rahbek 2011). The potential for large increases in temperature (e.g., 4°C+) (Betts et al. 2011) has major implications for forest ecosystems. Several studies have looked at climate change-driven shifts in the extent of humid forests (e.g., Zelazowski et al. 2011), while forests in subtropical regions, especially those in the Southern Hemisphere, have received relatively little attention. However, these forests are particularly vulnerable to climate change as they are likely to experience significant decreases in seasonal rainfall, as well as increases in temperature resulting from the subtropical zone expanding polewards (Cai et al. 2012). While previous research has focussed on species' polewards shifts in response to climate change, lack of accounting for lateral, east–west shifts could lead to miscalculation of its impact on biodiversity (VanDerWal et al. 2013): this has implications for both conservation and resource-use planning.

### Climate change in Australia

Australia's climate is uniquely highly variable, with most of the continent water-limited: the additive combination of temperature and rainfall change will govern climate change impacts here (McAlpine et al. 2009). During the last century, primarily since 1950, summer monsoon rainfall in northwest Australia has increased and rainfall in southern and eastern Australia has decreased (Smith 2004). Droughts have become correspondingly more severe (Nicholls 2004). Available moisture is projected to decrease, most strongly in the west, south, and central regions of Australia (CSIRO 2007), which will affect evaporation and evapotranspiration. A transition to a hotter and more drought-prone climate represents a major risk of Australia's ecosystems (Bennett et al. 2013), particularly for those without the capacity to recover or adapt (McAlpine et al. 2009).

### Trees and ecosystems

Trees will be among the first groups to be affected by climate change (Boardman 1994), as they are particularly vulnerable due to long regeneration times and generally short dispersal distances. These slow response times mean they will be unlikely to track climate change sufficiently rapidly to avoid high mortality rates (Solomon and Kirilenko 1997). Changes to forest and woodlands community composition and shifts in distribution, as a response to changes in climate, will have cascading effects for fauna and flora, and ecosystem services. Changes in the timing of fruit, seed, and flower availability could drive range shifts or mortality in species reliant on those resources (e.g., birds, bats, and invertebrates) (Parmesan 2006). Trees are a critical component of the hydrological cycle, with climate-related forest mortality resulting in a reduction of evapotranspiration and an increase in sensible energy fluxes (McAlpine et al. 2009; Pielke et al. 2011), with flow-on effects for water security (Mikkelson et al. 2013).

### Eucalypts

The eucalypt group reflects the unique character and diversity of the Australian flora and is globally exceptional in terms of its UNESCO World Heritage value. The eucalypt group is large (comprising *Eucalyptus, Angophora,* and *Corymbia* genera) and almost entirely restricted to Australian continent. There is a detailed understanding of individual eucalypt species' climatic requirements and ecophysiological tolerances, which can inform thermal ranges and drought tolerance (Wardell-Johnson et al. 1997; Brooker and Kleinig 1999). Climate change will act on species individualistically, and these responses may combine to trigger an ecosystem-level response. Hughes et al. (1996) innovative work established eucalypt species' thermal and moisture tolerance ranges for 819 eucalypt species, based on annual temperature and annual rainfall values at the point locations (of current distributions). The narrowness of the resulting ranges (e.g., more than 20% of species had a range of less than 1°C and <20% variation in rainfall), suggested that a large number of species would face conditions outside their current range and accordingly, large-scale changes in eucalypt species composition would follow. Further work has shown that changes in rainfall regimes are partially responsible for a historical switch from *Eucalyptus* forest and grassland to rainforest in some areas of Australia (Bowman et al. 2001; Hughes 2003) and that landscape variables such as soil and topography are also key determinants of species' distributions (Austin and Van Niel 2011).

We aimed to assess the vulnerability of species and species groups to climate change and predict the magnitude and direction of shifts in distribution. We applied a bioclimatic modeling approach to 108 eucalypt species. We examined the importance of seasonal and mean annual climate and compared the impacts of precipitation and temperature under different climate scenarios on species' climate space. This allowed us to identify which groups of species will be affected by climate change, how the impacts will differentially drive changes in distribution though shifts in suitable climate space, and what are the consequences for biodiversity.

## Methods

### Species distribution data

We used tree occurrence data from Australia's Virtual Herbarium (AVH) (extracted 2012), for *Eucalyptus* and *Corymbia* tree species, two of the three eucalypt genera. These were “cleaned,” which included careful consideration of the history of taxonomic naming for each species, removal of duplicate locations, and clarification and/or removal of spurious points. Species selection was based on Bureau of Meteorology climate classes (Fig. S1) in consultation with experts (including at the Queensland Herbarium and the University of Melbourne), and tree atlases (Brooker and Kleinig 1999), to identify appropriate species to use as representatives of climate region, community status (dominant/typical/endemic), and range breadth (wide/narrow/local). The 16 climate region groups were allocated to four broad geographical categories; tropical + equatorial, desert + open woodland, subtropical, and temperate (as in Table 1); the “wide range” group contained species whose distributions were not closely linked to any of the climate regions.

**Table 1:** AUC values for the model; training (75%) and test (25%) data, and two most significant contributing variables. The test AUC describes the fit of the model to the test data and gives the true predictive power of the model; AUC of 0.5 would be expected from a random model.

	Species (N)	Training AUC	Test AUC	Variable contribution to model	
Tropical + Equatorial					
Savanna – equatorial + tropical	3	0.99	0.99	Annual precipitation	Precipitation of coldest quarter
Savanna – tropical	12	0.97	0.95	Annual precipitation	Precipitation of coldest quarter
Tropical rainforest + savanna	4	0.99	0.99	Annual precipitation	Precipitation of coldest quarter
Grassland/open woodland –hot, winter drought	2	0.98	0.98	Precipitation of coldest quarter	Annual precipitation
Desert + open woodland					
Desert	4	0.97	0.97	Annual precipitation	Mean temperature of wettest quarter
Grassland/open woodland – hot, dry	5	0.98	0.97	Annual precipitation	Clay (%)
Grassland/open woodland – hot, summer drought	5	0.98	0.98	Precipitation of coldest quarter	Mean temperature of wettest quarter
Grassland/open woodland – warm, dry, summer drought	3	0.99	0.98	Mean temperature of wettest quarter	Precipitation of driest quarter
Subtropical					
Subtropical distinctly dry summer	5	0.99	0.99	Precipitation of coldest quarter	Mean temperature of wettest quarter
Subtropical distinctly dry winter, hot grassland/open woodland	2	0.98	0.97	Annual precipitation	Precipitation of driest quarter
Subtropical moderately dry winter, grassland/open woodland	4	0.98	0.97	Annual precipitation	Precipitation of driest quarter
Subtropical no dry season	5	0.99	0.99	Annual precipitation	Precipitation of driest quarter
Temperate					
Temperate distinctly dry summer	11	0.99	0.99	Mean temperature of wettest quarter	Precipitation of coldest quarter
Temperate no dry season hot/warm summer	7	0.97	0.96	Precipitation of driest month	Precipitation of driest quarter
Temperate no dry season warm summer	7	0.99	0.98	Max temperature of warmest month	Precipitation of driest quarter
Temperate no dry season, dry/mild/warm summer	11	0.99	0.98	Max temperature of warmest month	Precipitation of driest quarter
Wide range	18	0.93	0.92	Annual precipitation	Mean temperature of wettest quarter

### Climate predictors and scenarios

Climate variables were selected from the bioclimate dataset prepared for Australia, as part of the Wallace Initiative, at 5-km resolution (VanDerWal 2012). Test model runs using a subsample of our 108 selected species identified 6 of the 19 bioclimate variables as most correlated with current distributions. Although the significance of each predictor varied by species, the group of variables selected were most representative overall, and interactions between the climate variables and tree ecophysiology were also used to guide the selection. These were include: maximum temperature of the warmest month; mean temperature of the wettest quarter; annual precipitation; precipitation of the driest month; precipitation of the driest quarter; precipitation of the coldest quarter. The bioclimatic scenarios were available at ten-year time steps, from 2015 to 2085.

We selected two of the four Representative Concentration Pathway (RCP) scenarios: RCP8.5, a rising radiative forcing pathway resulting in 8.5 W m^−2^ by 2100, which reflects high levels of energy demand and greenhouse gas emissions without climate change policies and; RCP6, a stabilization-without-overshoot pathway to 6 W m^−2^ by 2100 (Moss et al. 2010), which corresponds to a peak in greenhouse gases by 2060 and then a reduction, driven by the global market for emissions reduction, for the rest of the century (Masui et al. 2011). The two scenarios, one mid-range and one extreme, reflect the most likely climate outcomes given the current level of mitigation activity.

### Soil/substrate predictors

Soil substrate is an important determinant of the distribution of eucalypt species, with individual species adapted to landscape-scale variations in soil structure and fertility (Austin and Van Niel 2011). Soil substrate predictors used were clay (median% clay content), pedality (soil structure), and SOLPAWHC (plant available water holding capacity of the solum, mm) (Williams et al. 2010). These were derived from the soil attributes compiled from the 1:2 M Atlas of Australian Soils based on their principle profile forms (PPF) (McKenzie et al. 2000; Australian Soil Resource Information System). Each attribute was calculated as the average of up to five PPFs per map unit, and also average-weighted by the depth of the A and B horizons where relevant. These 1-km resolution data were aggregated to 5-km cell resolution to align with the bioclimatic data. Fine-scale variation in topography and soils also influence species distribution; however, these high-resolution data are lacking at the continental scale. We utilized the 5-km cell soil and substrate data as they provide an useful overview of broad scale variation in edaphic conditions.

### Maxent modeling

Species distribution modeling enables prediction of changes in suitable climate space for species in response to climate change (e.g., Hijmans and Graham 2006). The Maxent (maximum entropy) algorithm derives the probability of presence or absence on a pixel-by-pixel basis and has the advantage over other species distribution models in that it requires presence-only data (see (Phillips et al. 2004, 2006) for detail). The probability output gives values between 0 and 1 and is calculated by minimizing the relative entropy between the two probability densities of the landscape covariates, with and without species presence (Elith et al. 2011). MaxEnt has been used widely for species distribution modeling in a conservation context and has been well-described elsewhere (e.g., Graham and Hijmans 2006; Adams-Hosking et al. 2012). In preparation for the analysis, we tested various Maxent parameterization options and selected those that performed best, that is, gave the best representation of the current distribution of the species without overfitting the model (see Merow et al. 2013).

Each species' distribution was modeled for current climate and three future projected climates: 2025, 2055, and 2085. This represented the projected range changes in distribution over most of the current century. A species' “suitable climate space” is derived from its modeled distribution. The model was created using 75% of the species' presence data, and the remaining 25% were used for testing the model's predictive strength. Area under the receiver operating curve or AUC values for training and testing and for jackknife analyses, and% contribution of each bioclimate or substrate predictor, were calculated for each species (e.g., Adams-Hosking et al. 2012). In order to estimate change in area (of climate space) and the direction of shift, the Maxent output logistic threshold was applied to give binary pixel occupation data (Phillips et al. 2006). Although using the threshold in this way means we discarded probability information from the model, these type of binary data are required for conservation planning – for reserve design, for example, – and also enable us to quantify potential gains and losses in climate space. These binary data were analyzed using R “raster” function (R Core Development Team 2008) to give metrics for persistence, loss of area, and gain of area. Species were analyzed individually and as groups (as in Table 1).

## Results

### Predictor variables

The key bioclimate predictor variable was annual rainfall. The two measures of seasonality (dry quarter precipitation, directly correlated with drought, and wet quarter temperature) were also very influential (Table 1). Other bioclimate and soil and substrate variables did not contribute significantly to the model. The training and test AUC values were uniformly high for all species models (Table 1). Temperature increases in spring and winter have historically been greater than for other times of the year (Hulme and Sheard 1999), which means that wet quarter temperature may become even more critical as a driver. Tree species were grouped by climate region (Table 1). For the “tropical + equatorial” climate region tree groups, tropical and equatorial savannas, and rainforest and hot grasslands/open woodlands, the most important bioclimatic variables were annual and coldest quarter precipitation. For the “desert + open woodland” climate region eucalypt species, annual and coldest quarter precipitation and mean temperature of the wettest quarter were the most important predictors. For the “subtropical” climate region tree groups, annual and driest quarter precipitation contributed most to the models and for the “temperate” climate region tree groups, driest quarter precipitation, mean temperature of the wettest quarter, and maximum temperature of the warmest month were most significant. For the widespread eucalypt species, annual precipitation was the key predictor variable (Table S1).

### Climate space shifts

**Figure 1:**
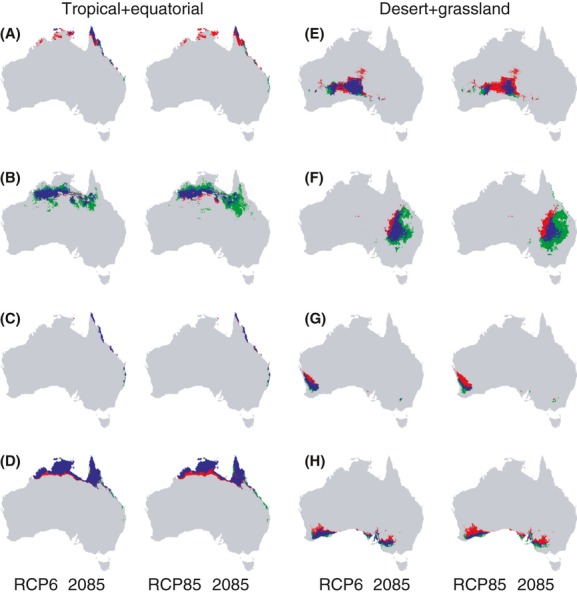
Representative species from each of the climate region groups in the “tropical + equatorial” class: (A) equatorial and tropical savanna (*Corymbia nesophila*); (B) tropical savanna (*Eucalyptus tetrodonta*); (C) tropical rainforest + savanna (*E. pellita*); (D) grassland/open woodland – hot, winter drought (*E. limitaris*), and the “desert + open woodland” class: (E) desert (*E. glomerosa*); (F) grassland/open woodland – hot, dry (*E. ochrophloia*); (G) grassland/open woodland – hot, summer drought (*E. stowardii*); (H) grassland/open woodland – warm, dry, summer drought (*E. cylindriflora*). The blue areas indicate no change in occupied climate space, the red shading indicates loss of climate space, and the green shading climate space gain. Species in the “tropical + equatorial” groups all showed range contractions under both climate scenarios. These contractions were from the west and southwest of their ranges, while there was some gain in the north and northeast of their ranges, and some species also expanding to the south. Species in the “desert + open woodland” groups showed contraction of the northern parts of their ranges. The “grassland/open woodland – hot, dry” group species lost a large part of their suitable climate space with only some fragments remaining under the most extreme scenario. For the “grassland/open woodland – warm, dry, summer drought” group, there was a shift in suitable climate space southwards.

**Figure 2:**
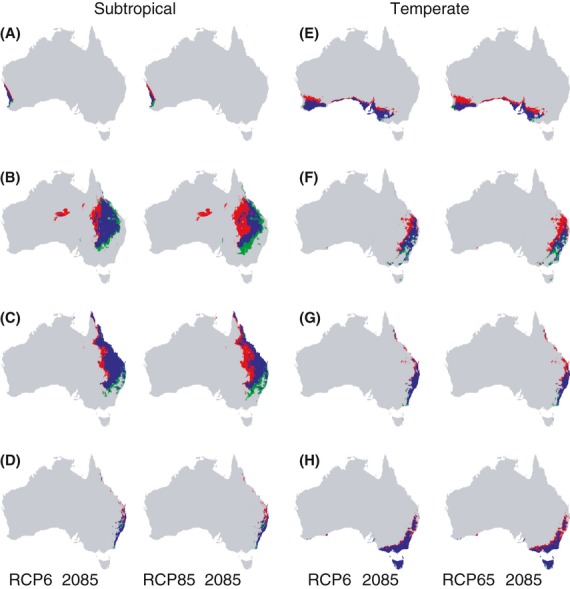
Representative species from each of the climate region groups in the “subtropical” class; (A) subtropical distinctly dry summer (*Eucalyptus todtiana*); (B) subtropical distinctly dry winter, hot grassland/open woodland, tropical (*E. thozetiana*); (C) subtropical moderately dry winter, grassland/open woodland, tropical (*E. exserta*); (D) subtropical no dry season (*E. pilularis*), and the “temperate” class; (E) temperate distinctly dry summer (*E. phenax*); (F) temperate no dry season, hot/warm summer (*E. conica*); (G) temperate no dry season, warm summer (*E.saligna*); (H) temperate no dry season, dry/mild/warm summer (*E. pauciflora*). The blue areas indicate no change in occupied climate space, the red shading indicates loss of climate space, and the green shading climate space gain. Species in the “subtropical” groups (which also includes some distributions in Western Australia which fall in the “subtropical distinctly dry summer” climate region) exhibited an overall shift southward. Species in the “temperate” groups all showed losses in the north/northwest of their ranges. There was some indication of expansion into the south of the range for three of the groups, but the “temperate no dry season, dry/mild/warm summer” group was already at the southern extent of the continent, with no option to expand further south.

**Figure 3:**
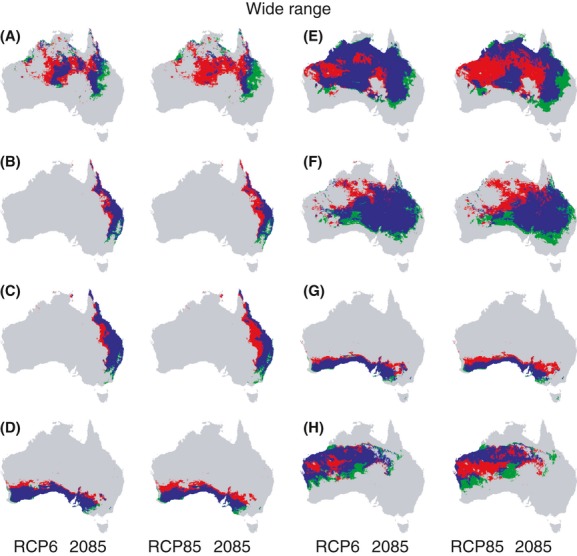
Sample species from the “wide range” climate region groups: (A) *Corymbia aparrerinja*; (B) *C. trachyphloia*; (C) *Eucalyptus crebra*; (D) *E. oleosa*; (E) *C. terminalis* (F) *E. coolabah*; (G) *E. gracilis*; (H) *E. victrix*. The blue areas indicate no change in occupied climate space, the red shading indicates loss of climate space, and the green shading climate space gain. The wide range species all showed an overall loss of suitable climate space, broadly in the north or west of their range, and some expansion south or southeast of their current range.

**Figure 4:**
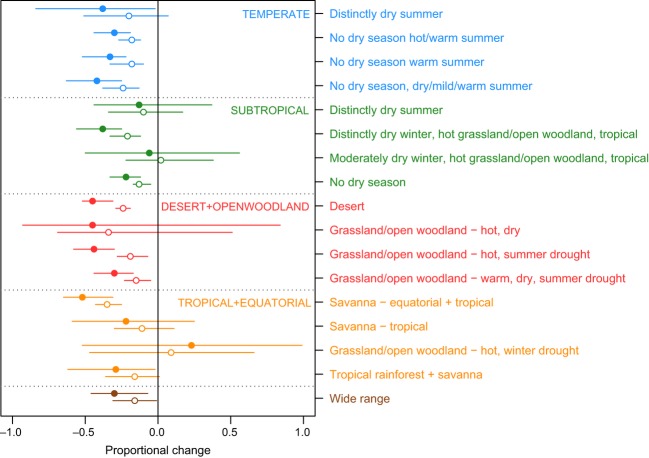
Proportional (%) pixel losses and gains by climate regional group, calculated from the 2085 time step for both scenarios. For each species, pixel losses and gains were calculated as a proportion of the current number of pixels occupied. The lines with solid circles represent group ranges and means for the RCP85 scenario; the lines and hollow circles show the group ranges and means for the RCP6 scenario.

Thresholds were applied to the probability distributions for each species in order to estimate changes in pixel occupation: the changes in suitable climate space were derived from the difference between a species' modeled current and modeled future climate space. As with the probability distributions (Fig. S2), the change was progressive. An example of distribution shifts under the RCP6_2085 and RCP85_2085 scenarios is given for each group (Figs. 1–3). Most climate region groups lost climate space under both scenarios, but more markedly under the RCP8.5 scenario (the RPC6_2085 and RCP8.5_2055 scenarios were comparable in their degree, and direction of impact on species distribution changes). However, the grassland/open woodland – hot, winter drought group indicated gains in climate space under both scenarios, and the subtropical, moderately dry winter, grassland/open woodland group gained some space under the RCP6 scenario. The climate region group predicted to lose the most climate space was the savanna – equatorial + tropical group, while at the larger geographical scale, the “desert + open woodland” group lost the most space proportionally; >40% (Fig. 4; Table S1).

**Figure 5:**
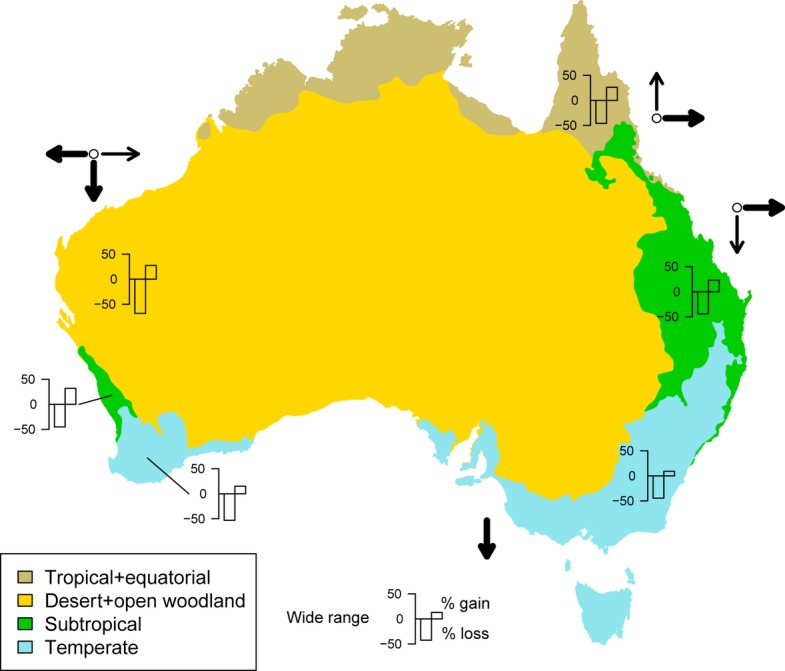
Large-scale shifts in climate space under both RCP6 and RCP85 scenarios. Tree climate regions were broadly based on the Bureau of Meteorology climate classification, as above. The arrows indicate the overall direction and magnitude of climate space shift for the four general climate regions. The bar charts show percentage gain and loss for each of the four regions and the “wide range” group; “temperate distinctly dry summer” (light blue, left), and “subtropical – distinctly dry summer” (green, left) are shown separately.

Analysis of the direction of range contraction and expansion, or climate space shift, from both probability and threshold data, indicated that species ranges in the north will shift further north and east, species ranges in the east will shift further east and south, species ranges in the “desert + open woodland” class will shift primarily south and west, and species ranges in the “temperate” class will shift south (Fig. 5). Large areas of central Australia became climatically unsuitable for eucalypts under either scenario. Many of the species modeled indicated shifts toward the southern and eastern coasts, leaving no option for range expansion. Of the 108 species modeled, all showed some gain and some loss of suitable climate space pixels when thresholds were applied to the output models. Ten species showed greater gain than loss. Only four of these – all of them savanna or grassland/open woodland species – showed a > 10% gain (Table S1).

## Discussion

The overall prognosis for eucalypt species is one of increasing environmental stress and potentially large distributional shifts under climate change. The extensive summer drying and more frequent droughts in the interior of Australia (Meehl et al. 2007) will result in the large predicted losses of suitable climate space for the “desert + open woodland” category. Physiological stress on species in these climatic groups will be intensified as precipitation seasonality increases, and a reduction in relative humidity and increase in evapotranspiration will combine to increase moisture stress (CSIRO 2007). There has been some discussion of the possible effects of CO_2_ fertilization, which may ameliorate moisture stress by promoting water-use efficiency through the mechanism of reduced stomatal conductance (Wullschleger et al. 2002), thereby counteracting the potential water stress problems caused by conditions becoming warmer and drier (Hughes 2003). This is a plausible mechanism, but it is not known what the limits to water-use efficiency improvement are, and at what point the decrease in available moisture would override all CO_2_ fertilization effects.

### Patterns and drivers

For the species in the “subtropical” and “temperate” groups, precipitation of the driest quarter was one of the key predictor variables. With the predicted increase in evaporation and decrease in rainfall in eastern Australia (Suppiah et al. 2007), seasonal water stress (as drought) is likely to increase, further reducing suitable climate space for these species. Rainfall is also projected to decrease within 400 km of western and southern coasts and in subtropical areas, and rainfall seasonality is predicted to increase in tropical and central areas (Suppiah et al. 2007). Seasonal moisture availability is a key limiting factor in forest systems (Butt et al. 2008) and could provide an ecological tipping point for *Eucalyptus* forest persistence in these climate regions (cf. Malhi et al. 2009). In addition to changing climate influencing species and ecosystems, changes in ecosystem composition and forest cover could also impact on local hydrology and climate, further exacerbating environmental stress (McAlpine et al. 2009 and references therein).

The interconnection between heat and moisture is critical for species distributions. Heat stress can be ameliorated by moisture availability, while moisture stress is exacerbated by high temperatures (see Williams et al. 2013) for impacts on forests in the southwest USA). Many of the species in the “tropical + equatorial” group are savanna species, highly sensitive to heat stress and probably at the limit of their temperature tolerance (Dunlop et al. 2012). Projected increases in rainfall seasonality and drought occurrence are reflected in the general loss of species' climate space from the west and southwest of their ranges.

### Environmental tolerance ranges

Previous modeling work indicates that more than half of all eucalypt species have a climate space range of less than ±3°C of annual mean temperature, and for a quarter of species the range is less than ±1°C of annual mean temperature (Hughes et al. 1996). About a quarter of species have a precipitation range of ±20% of current climate (Hughes et al. 1996). Although the environmental tolerance of many species is probably wider than the climate space they currently occupy, current distributions of species represent species' adaptation to local conditions and dispersal limitations. Previous extreme drought events have caused large-scale eucalypt dieback, which suggests that some eucalypts, for example, savanna species in northern Queensland, already exist at the limits of their climatic tolerance range (Fensham and Holman 1999). Such species would therefore have no capacity to persist if environmental tipping points, such as length of drought or amount of seasonal rainfall, are reached. Dry areas are becoming drier and wet regions wetter, in terms of annual rainfall, while increasing seasonality also means that dry seasons are becoming drier in drier areas and wet seasons are becoming wetter in wetter areas (Chou et al. 2013). This intensification of rainfall seasonality may significantly impact drought and flood frequency (Chou et al. 2013); droughts are projected to increase in frequency and severity (Meehl et al. 2007). The physiological stress driven by the key interaction between increasing temperatures and increasing moisture seasonality will be further exacerbated by the predicted rise in evapotranspiration, which will be greatest in the north and east of the continent (CSIRO 2007). The steady increase in evapotranspiration deficit over recent decades has already led to significant dieback of the iconic red river gum (*Eucalyptus camaldulensis*) across the Murray–Darling Basin (Mac Nally et al. 2011).

Historical and fossil record data demonstrate that some species have persisted in their current location through previous changes in climate (which were outside their current climate range) (Hopper 2009). This may be a function of topographic diversity, which could enable long-term persistence as species need to move shorter distances to track climate change, or provide habitat heterogeneity. Alternatively, these species may have a larger capacity to buffer extremes of climate, through physiological resistance or phenotypic plasticity, and may continue to persist under future climate. Many *Eucalyptus* species' ranges are narrow and closely linked with local environmental drivers, such as soil characteristics (Hughes 2003; Austin and Van Niel 2011). Microhabitats, by providing an environment within the thermal or moisture tolerance range of the species, can offer protection against heat and moisture stress, and so enable the persistence of individuals or groups of individuals. For example, genetic evidence suggests that some eucalypt species may have persisted in mesic microhabitats in drier, colder climates and recolonized drier slopes when conditions improved (Pollock et al. 2013). It is difficult to postulate whether this would be sufficient to maintain species in their current climate space or act only as a delay on the climate change-driven decline. These microhabitats could act as refuges by providing a different habitat, and thus enable the survival (for a time) of species or ecosystems differing from those of the surrounding area (Dunlop et al. 2012).

### Range shifts

The modeled shifts in range are east–west as well as north–south, reflecting movement away from the continental interior. In general, only poleward shifts are taken into consideration in terms of species distribution shifts (VanDerWal et al. 2013). As with the bird species modeled (VanDerWal et al. 2013), eucalypt (species and groups) responses to increasing aridity, as a result of changes in rainfall and temperature, suggest multidirectional shifts in distribution. With the exception of the temperate climate region species, for all of the climate region groups modeled, east–west shifts were more significant than north–south shifts.

Due to fine-scale landscape heterogeneity, the forecast shifts will not be uniform but vary according to local environmental conditions. One anticipated trend is ecosystem-scale thinning of canopy species, but it is unknown whether increased water-use efficiency (in response to elevated CO_2_ levels) may buffer this change in structure, or act to promote shrub layer growth (Dunlop et al. 2012).

Other landscape drivers, such as fire, are similarly forecast to alter in frequency and intensity, and land use and land-use change also interact synergistically with change in climate variables (e.g., land/forest clearance, grazing regimes). Species interactions and invasive species behavior will play a role in changes in ecosystem composition and structure. Assuming that understory structural changes are largely driven by management, while overstory and dominant eucalypts are climatically controlled (Fensham and Holman 1999), changes in community composition and forest structure are likely happen at different rates for different forest strata.

### Conservation, carbon stocks, and restoration

The large predicted shifts in distribution will have major consequences for conservation of eucalypt-dominated ecosystems and all related/dependent fauna and flora. Distribution shifts will result in the absence of particular species in some ecosystems, the disappearance of entire ecosystems, or the creation of novel ecosystems – these will all affect the persistence of associated species and disrupt complex existing interspecific interactions in unpredictable ways. Conservation planning focussing, for example, on woodland-dependent species, will have to also consider the impact of climate change on the trees of that particular ecosystem and whether or not the woodland will persist.

In Australia, there are several programmes aimed at restoration of native vegetation from cleared areas in order to promote carbon sequestration and storage, such as the Carbon Farming Initiative (http://www.climatechange.gov.au/reducing-carbon/carbon-farming-initiative), which promotes carbon storage through sustainable land management practices and landscape restoration. Carbon storage (and, therefore, climate change mitigation) is a key ecosystem function for forests, especially significant as forests globally hold more carbon than the atmosphere (Pan et al. 2011). However, even proportionally small changes in woody vegetation and forest composition and structure can have large implications in terms of carbon storage and stocks as the land area of Australia is so large. Potential changes in climate space suitability should therefore be taken into account in carbon stock management schemes. Investment in carbon storage may not deliver the predicted benefit under recurring droughts and tree dieback.

### Global significance

Recent literature predominantly focuses on northern hemisphere predictions of climate change-related range shifts in temperate and boreal regions; however, our paper addresses the implications for many southern regions, for example, savannas with seasonally variable rainfall, which occur widely across Africa, South America as well as Australia. The analysis presented here provides further evidence that long-lived tree species are not capable of tracking rapidly changing climate, which has global implications given the projected magnitude of changes in climate, and the extent and importance of forest, woodland, and tree cover across most regions of the world. Changes in both mean climate and climate extremes will drive major shifts in many forest and woodland ecosystems, especially in hotter and drier regions such as the Chaco and Cerrado of South America, already under pressure from land-use change (Killeen et al. 2008). These changes will vary regionally and will have cascading consequences for the functioning of ecosystems and the persistence of their dependent fauna.

The UN Rio + 20 Conference on Sustainable Development agreed on a global target of restoration of 150 million ha of disturbed and degraded land by 2020 (Menz et al. 2013). However, if areas which have been set aside for restoration are no longer climatically suitable, this casts doubt on the benefit of restoration planning in these areas, establishing trees in such landscapes will be extremely difficult under changing climate conditions. With the global emphasis on habitat conservation and restoration, and now threatened ecosystems (IUCN), our findings demonstrate that planning restoration and conservation activity without considering patterns and directions of range shifts would incur a high risk of failure. This is globally pertinent, with large financial implications and consequences for biodiversity.

## Supporting Information

**Figure S1.** Bureau of Meteorology climate classes and subclasses on which the modeled tree species groups are based.

**Figure S2.** Modeled climate space probability distribution for the three time steps and two scenarios for a “wide range” species (*E. crebra*). The greener areas show a higher probability of presence. The Maxent models showed a trend of decreasing probability of presence through time for both scenarios and for most species. For widespread species, probability of presence broadly declined in the west or north and increased in the south or east.

**Table S1.** Proportional (%) pixel losses and gains by climate regional group calculated from the 2085 time step for both scenarios. For each species, pixel losses and gains were calculated as a proportion of the current number of pixels occupied. The group% loss and gain is the arithmetic mean of the species losses and gains within the group.
