# Supplementary material for: Eucalypts face increasing climate stress
Source: Ecol Evol. 2013 Nov 12;3(15):5011–22. doi: 10.1002/ece3.873 (PMC3892364; doi:10.1002/ece3.873)
Supplement: Supplementary file 1 [file ece30003-5011-SD1.docx]

**Table S1:** Proportional (%) pixel losses and gains by climate regional group, calculated from the 2085 time step for both scenarios. For each species, pixel losses and gains were calculated as a proportion of the current number of pixels occupied. The group % loss and gain is the arithmetic mean of the species losses and gains within the group.

|  |  | RCP6_2085 |  | change | RCP85_2085 |  | change |
| --- | --- | --- | --- | --- | --- | --- | --- |
|  |  | **group loss** | **group gain** |  | **group loss** | **group gain** |  |
| **tropical+equatorial** | **Savanna - equatorial+tropical** | 39.52 | 4.79 | -34.73 | 60.12 | 8.55 | -51.58 |
|  | **Savanna - tropical** | 17.32 | 6.15 | -11.17 | 32.53 | 10.13 | -22.40 |
|  | **Grassland - hot, winter drought** | 40.59 | 50.06 | 9.47 | 53.79 | 77.25 | 23.45 |
|  | **Tropical rainforest + savanna** | 21.69 | 5.62 | -16.08 | 37.35 | 8.64 | -28.71 |
| **desert+openwoodland** | **Desert** | 38.00 | 14.07 | -23.93 | 65.56 | 20.78 | -44.77 |
|  | **Grassland/open woodland - hot, dry** | 52.36 | 18.36 | -34.01 | 74.23 | 29.12 | -45.11 |
|  | **Grassland/open woodland - hot, summer drought** | 39.63 | 21.00 | -18.62 | 73.49 | 29.68 | -43.81 |
|  | **Grassland/open woodland - warm, dry, summer drought** | 32.70 | 18.09 | -14.61 | 61.34 | 30.94 | -30.40 |
| **subtropical** | **Subtropical distinctly dry summer** | 29.19 | 19.20 | -9.99 | 44.87 | 31.79 | -13.07 |
|  | **Subtropical distinctly dry winter, hot grassland/open woodland, tropical** | 31.98 | 10.59 | -21.38 | 55.51 | 17.66 | -37.85 |
|  | **Subtropical moderately dry winter, grassland/open woodland, tropical** | 20.99 | 22.79 | 1.80 | 41.67 | 35.95 | -5.72 |
|  | **Subtropical no dry season** | 22.86 | 9.91 | -12.95 | 38.56 | 16.69 | -21.87 |
| **temperate** | **Temperate distinctly dry summer** | 31.68 | 11.48 | -20.20 | 53.04 | 15.28 | -37.76 |
|  | **Temperate no dry season hot/warm summer** | 26.04 | 7.79 | -18.26 | 42.37 | 12.73 | -29.63 |
|  | **Temperate no dry season warm summer** | 23.43 | 5.34 | -18.10 | 40.86 | 8.16 | -32.70 |
|  | **Temperate no dry season, dry/mild/warm summer** | 24.18 | 0.60 | -23.57 | 42.49 | 0.51 | -41.98 |
| **everywhere** | **wide range** | 24.04 | 8.42 | -15.63 | 42.53 | 12.76 | -29.77 |
