# Supplementary figures and images for: Eucalypts face increasing climate stress
Source: Ecol Evol. 2013 Nov 12;3(15):5011–22. doi: 10.1002/ece3.873 (PMC3892364; doi:10.1002/ece3.873)

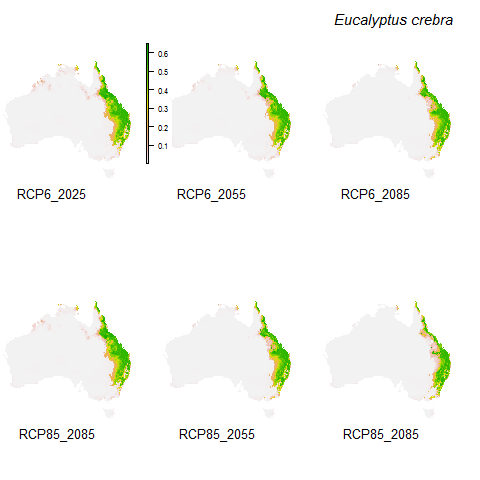

Supplement: Supplementary file 2 [file ece30003-5011-SD2.tif]

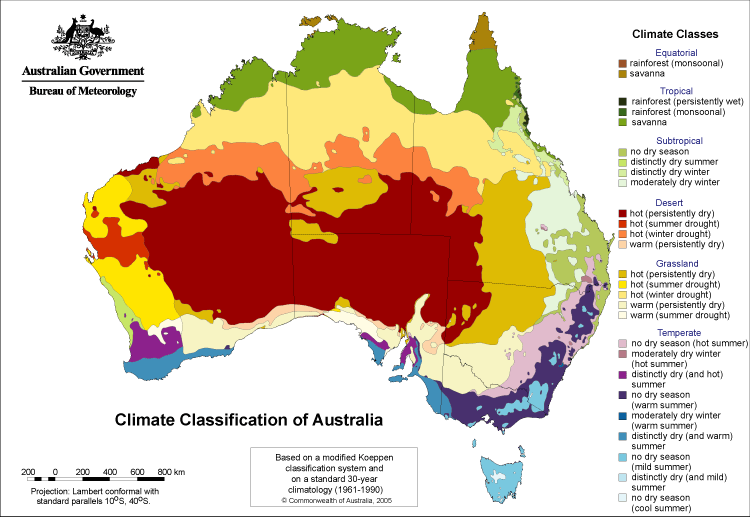

Supplement: Supplementary file 3 [file ece30003-5011-SD3.tiff]
